# Evaluation of the value of preoperative CYFRA21-1 in the diagnosis and prognosis of epithelial ovarian cancer in conjunction with CA125

**DOI:** 10.1186/s13048-019-0587-0

**Published:** 2019-11-25

**Authors:** Chunjing Jin, Minfeng Yang, Xueqiao Han, Haidan Chu, Yan Zhang, Meihong Lu, Zhonghui Wang, Xinxin Xu, Wenwen Liu, Feng Wang, Shaoqing Ju

**Affiliations:** 1 Laboratory Medicine Center, Affiliated Hospital of Nantong University, 20 Xisi Road, Nantong, 226001 China; 2 Teaching and Research Office of Medical Laboratory, Suzhou Vocational Health College, 1 Kerui Road, Huqiu District, Suzhou City, 215004 Jiangsu Province China; 3 Clinical medical laboratory, Jiangsu University, 301 Xuefu road, Jingkou District, Zhenjiang city, 212013 Jiangsu Province China

**Keywords:** CYFRA21-1, Diagnosis, Prognosis, Epithelial ovarian cancer

## Abstract

Growing evidence indicates that the tumor biomarker cytokeratin 19 fragment (CYFRA21-1) is significant for a variety of cancers. However, its role in epithelial ovarian cancer (EOC) has rarely been reported. In this study, a receiver operating characteristic (ROC) curve was utilized to estimate the diagnostic efficiency of CYFRA21-1. The correlation between the CYFRA21-1 level and prognosis was analyzed by Kaplan-Meier survival analysis and univariable and multivariable analyses. The relationship between serum CYFRA21-1 levels and different clinicopathological variables was also analyzed. At the same time, the standard serum marker cancer antigen 125 (CA125) was measured. The results demonstrated that CYFRA21-1 expression was significantly increased in EOC compared with expression in benign ovarian diseases and healthy controls, which was similar to CA125 (*P* < 0.001). CYFRA21-1 expression was positively correlated with CA125 (r = 0.201; *P* = 0.0032). CYFRA21-1 expression was significantly correlated with lymph node metastasis and ascites (*P* < 0.001). Furthermore, the median survival time of EOC patients with high CYFRA21-1 expression was 42 months, compared with 54 months in the low CYFRA21-1 expression patients by Kaplan-Meier analysis (*P* < 0.05), while the high and low CA125 expression groups had no difference in median survival time. Univariate and multivariate analyses indicated that CYFRA21-1 was a poor prognostic factor associated with overall survival (OS), while CA125 was not. Our study indicates that CYFRA21-1 acts as a good complementary diagnostic biomarker and may be superior to CA125 as a prognostic indicator in EOC.

## Introduction

Ovarian cancer is the third most common gynecologic malignancy and has a high mortality. Due to tissue and anatomy characteristics, the symptoms of ovarian cancer are easily ignored by patients and are not easily distinguished from other benign ovarian diseases. Most patients are diagnosed at stage III or IV. The 5-year survival rate of these patients is lower than 30% [1–3], while the 5-year survival rate is over 90% for patients with stage I [4]. Epithelial ovarian cancer (EOC) is the most common type of ovarian cancer, as it accounts for 80–90% of cases [5], which is further divided into serous, mucinous, endometrioid, clear cell and other mixed or rare subtype. Among them, the serous subtype is the most aggressive.

Currently, trans-vaginal ultrasonography (TVUS) and pelvic examination are the “gold standard” for detecting EOC [1]. However, complicated invasive procedures have scared patients off, and TVUS is seldom available in small local hospitals. At present, CA125 is the most important biomarker for EOC in the clinic, and it is also the most important serum marker for assessing therapy and relapse regarding EOC [6, 7]. However, it has many disadvantages, such as a low detection rate for early diagnosis and limited specificity [8]. Besides, it can be detected with high levels in benign gynecological diseases, for example, pelvic infections, fibroids and endometriosis [9, 10].

Cytokeratin 19 fragment (CYFRA21-1) was first reported in 1993 and is a fragment of cytokeratin subunit 19 [11]. High expression of CYFRA21-1 has been investigated in squamous cell carcinoma and is usually used as a tumor biomarker in patients with non-small cell lung cancer [12]. Keratin, which plays an important role in the structural stabilizers of epithelial cells, acts as a biomarker in the differentiation of epithelia [13]. We hypothesize that there is a correlation between CYFRA21-1 and EOC. In the present study, we explored CYFRA21-1 expression in EOC and evaluated the value of its clinical application.

## Materials and methods

### Study population

After receiving the approval of the Institutional Review Board for the medical records, we retrospectively analyzed data in tumor registry and pathology databases. A total of 203 EOC patients who underwent surgical treatment, 341 serum samples from healthy women and 150 serum samples from benign ovarian diseases were recruited for this study. The study was approved by the Ethics Committee of Affiliated Hospital of Nantong University.

### Laboratory data collection

All patients were confirmed by pathological diagnosis on surgical specimens, and all subjects had serum CYFRA21-1 and CA-125 levels recorded within 1 week before operation. None of the patients had received neoadjuvant chemotherapy or radiation prior to surgery. The following clinicopathological data were also collected: age at diagnosis, histological type, menopausal status, tumor grade and disease stage. The neoplasms were analyzed by histology and grade classified according to World Health Organization criteria. The grade was based on the Silverberg standard, and the stage was based on International Federation of Gynecology and Obstetrics (FIGO) standards. The chemiluminescent microparticle immunoassay (CMIA) (Abbott Diagnostics Division, Chicago, USA) was used to measure serum levels of CYFRA21-1 and CA-125.

### Follow-up

Follow-up with all EOC patients occurred by telephone once every 3 months in the first 2 years and every 6 months after that. The duration of overall survival (OS) was calculated as the time from diagnosis to death or the last follow-up time for the living patients.

### Statistical analysis

The levels of serum CA-125 and CYFRA21-1 are expressed as the median ± IQR. The Mann-Whitney U-test was used for comparisons with controls. Receiver operating characteristic curves (ROC) were generated to evaluate the diagnostic value. Sensitivity, specificity, positive predictive value (PPV), and negative predictive value (NPV) were supplied by recommended cut-offs. OS was analyzed by Kaplan-Meier analysis, and survival curves were examined by the log-rank test. Univariate and multivariate analyses were performed to determine which factors predict OS. Analyses were all performed using SPSS software version 20.0 (SPSS Inc., Chicago, IL). *P* < 0.05 was considered statistically significant.

## Results

### Patient characteristics

A total of 203 epithelial ovarian patients met the inclusion criteria. The mean duration of follow-up was 32 months. The mean age of the patients was 54.6 years. Their characteristics are described in Table 1. A total of 203 patients with EOC aged from 26 to 93 years (average: 55 years), 341 healthy women aged from 19 to 87 years (average: 54 years) and 150 benign ovarian tumor samples from patients aged from 20 to 81 years (average: 50 years) were obtained. There was no significant difference in age among the patients in these groups.

**Table 1:** Baseline characteristics of 203 epithelial ovarian cancer patients (EOC)

Variables	Mean (range or %)
Mean age at diagnosis (years)	54.6 (26–93)
Age	
< 55 years	111 (54.68)
≥ 55 years	92 (45.32)
Menopause	
Yes	115 (56.65)
No	88 (43.35)
Tumor size	
< 5 cm	76 (37.44)
≥ 5 cm	127 (62.56)
Subtype	
Serous	144 (70.94)
Mucinous	16 (7.88)
Endometrioid	24 (11.82)
Clear cell	15 (7.39)
M/R	4 (1.97)
Grade	
1	34 (16.75)
2	37 (18.23)
3	132 (65.02)
Stage	
I + II	102 (50.25)
III + IV	101 (49.75)
Lymph node	
Yes	65 (32.02)
No	138 (67.98)
Ascites	
Yes	29 (14.29)
No	174 (85.71)
Follow-up (months)	32 (3–60)

### Serum CYFRA21-1 is highly expressed in EOC

**Fig. 1:**
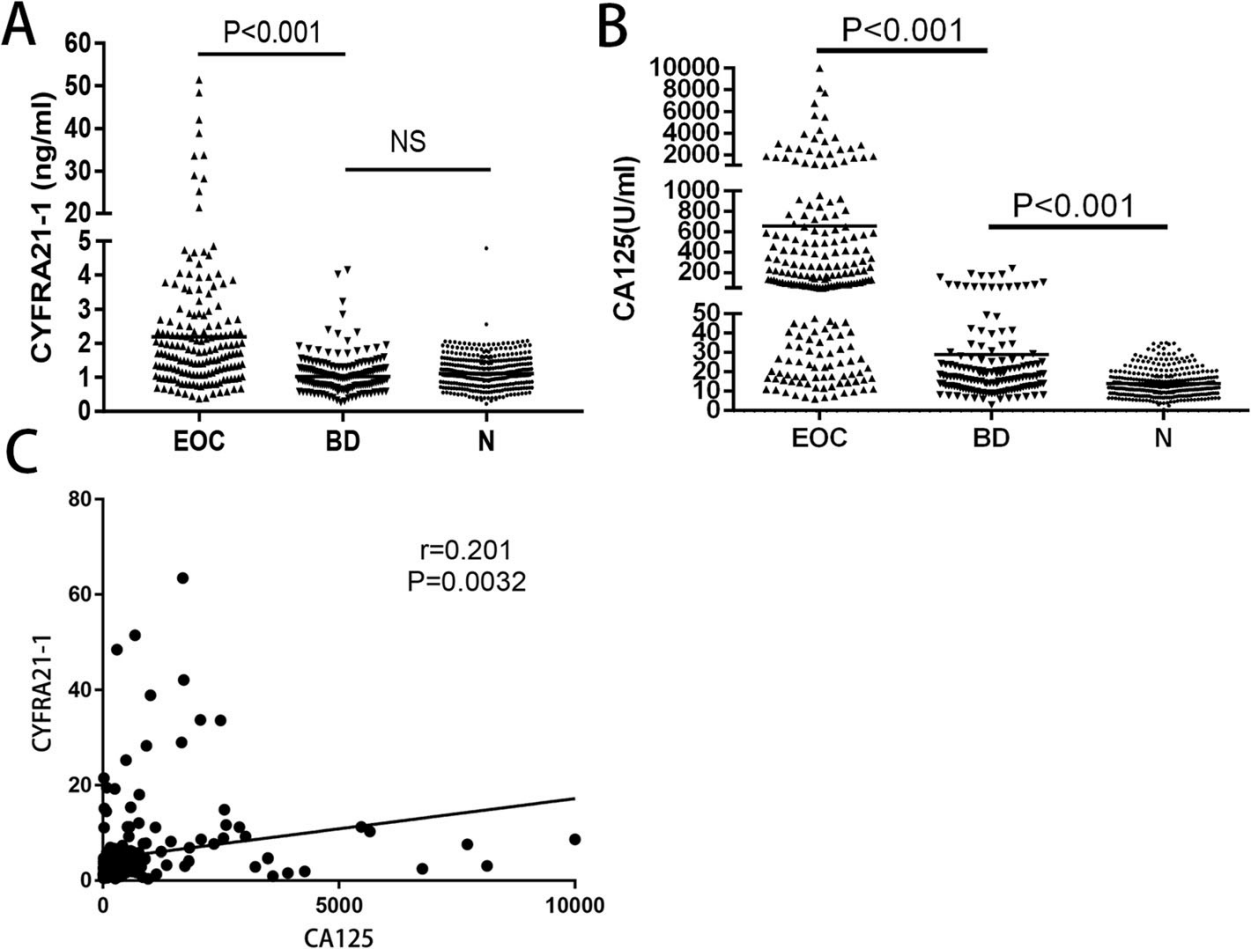
**a** Scatter plots of CYFRA21-1 expression in serum from N, BN and EOC. **b** Scatter plots of CA125 expression in serum from N, BN and EOC. **c** Relationships between CYFRA21-1 and CA125. Normal controls (N), benign diseases (BN), epithelial ovarian cancer patients (EOC)

It is worth mentioning that preoperative levels of CYFRA21-1 were significantly higher in EOC patients than those in the benign group and the normal control (*P* < 0.001), but there was no significant change in CYFRA21-1 levels between the benign group and the normal control (Fig. 1a). Preoperative levels of CA125 were significantly higher in EOC patients than those in the benign group and the normal control (*P* < 0.001), and there was a significant difference between the benign group and the normal control (*P* < 0.001). It proved that CA125 is a better indicator for diagnosing EOC (Fig. 1b). In addition, we found that preoperative CYFRA21-1 was positively correlated with CA125 in EOC patients (*r* = 0.201; *P* = 0.0032) (Fig. 1c).

### Evaluation of the diagnostic value of serum CYFRA21-1 for EOC

**Fig. 2:**
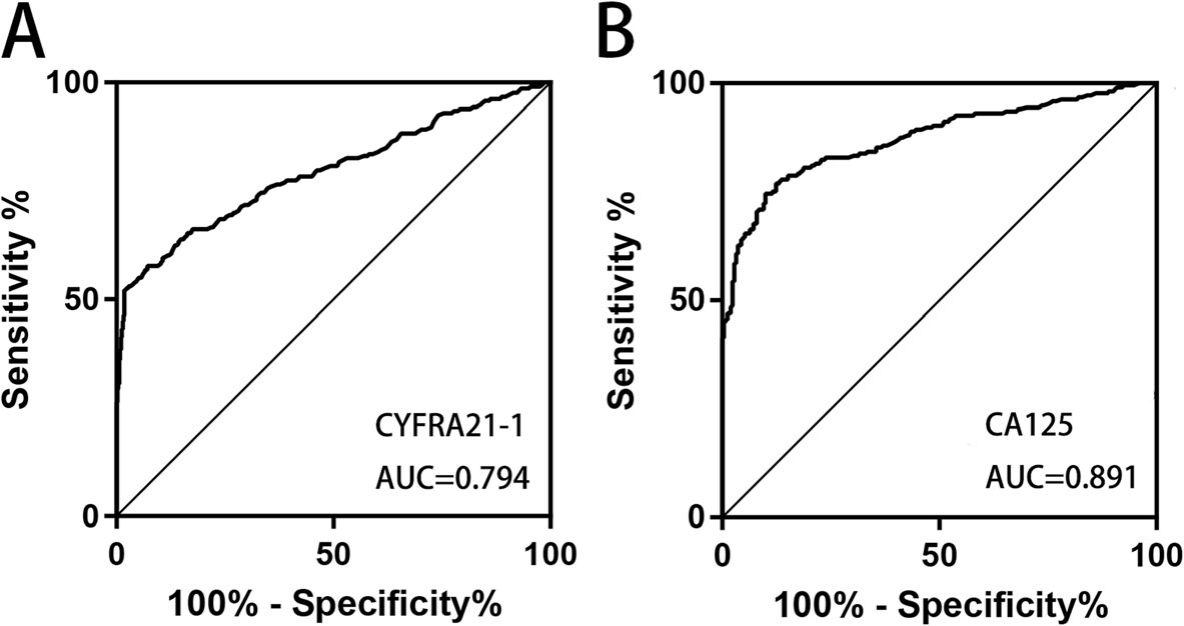
**a** Receiver-operating characteristic curve of CYFRA21-1. **b** Receiver-operating characteristic curve of CA125

Youden’s index was calculated using the receiver operating characteristic curve (ROC) to obtain the optimal cutoff values (Fig. 2a & Fig. 2b). When a CYFRA21-1 value was 2.08 ng/mL, the maximum Youden’s index existed. Therefore, the cutoff value of CYFRA21-1 for diagnosing EOC was set at 2.08 ng/mL (51.6% sensitivity and 98.2% specificity). The area under the curve for CYFRA21-1 was 0.794 with a 95% confidence interval (0.753, 0.835). By that analogy, the cutoff value used for CA125 was 35 U/ml (74.2% sensitivity and 94.9% specificity). The area under the curve for CA125 was 0.891 with a 95% confidence interval (0.860, 0.921).

Next, a combined analysis of CYFRA21-1 and CA125 was performed using a tandem model of the two markers. Table 2 shows that the sensitivity of this panel was 82.8%, which was distinctly superior to any biomarker alone. At the same time, the NPV was very high in the panel (85.7%) but at the cost of a moderately low PPV (75%). These findings suggest that we could consider CYFRA21-1 measurements in the diagnosis of EOC to increase specificity.

**Table 2:** Diagnostic efficiency of the CYFRA21-1 and CA125 combined analysis

	SEN	SPE	PPV	NPV
CYFRA21-1	51.6%	98.2%	93.3%	79.4%
CA125	74.2%	94.9%	95.2%	74.1%
CYFRA21-1 + CA125	82.8%	55.7%	75%	85.7%

*SEN* Sensitivity, *SPE* Specificity, *PPV* Positive predictive value, *NPV* Negative predictive value

### Comparison of clinical and pathologic characteristics

Next, we analyzed the expression of CYFRA21-1 in different subgroups of EOC patients with respect to clinicopathologic parameters such as age, menopause, tumor size, subtype, and grade **(**Table 3**)**. We found that CYFRA21-1 was correlated with grade, stage, lymph node and ascites (*P* < 0.05) in EOC patients. As is known, these factors are risk factors leading to poor outcome. Therefore, we believe that CYFRA21-1 has something to do with OS.

**Table 3:** Correlation between clinical parameters and CYFRA21-1

Clinical characteristics	No.	CYFRA21-1 Median (IQR25–75)	*P* value
All	203	5.26 (1.27–5.24)	
Age			0.062
< 55 years	111	1.75 (0.97–3.85)	
≥ 55 years	92	2.68 (1.58–6.67)	
Menopause			0.059
Yes	115	1.82 (1.13–4.66)	
No	88	2.34 (1.42–5.96)	
Tumor size			0.8714
< 5 cm	76	1.96 (1.22–5.79)	
≥ 5 cm	127	2.20 (1.29–5.03)	
Subtype			
Serous	144	2.43 (1.40–6.01)	0.0547
Mucinous	16	1.55 (0.96–1.89)	
Endometrioid	24	1.89 (1.09–4.38)	
Clear cell	15	1.49 (0.99–2.51)	
M/R	4	1.97 (1.71–2.25)	
Grade			0.000*
1	34	0.99 (0.79–1.76)	
2	37	1.41 (1.06–2.11)	
3	132	2.795 (1.38–11.12)	
Stage			0.000*
I	33	4.05 (0.86–3.99)	
II	69	2.94 (1.08–2.43)	
III	52	9.29 (1.17–9.76)	
IV	49	8.31 (2.24–10.72)	
Lymph node			0.000*
Yes	65	5.07 (2.23–11.80)	
No	138	1.33 (0.77–2.67)	
Ascites			0.000*
Yes	29	5.79 (1.97–7.90)	
No	174	1.50 (0.80–4.07)	

*P < 0.05

### Dynamic analysis of serum CYFRA21-1 in EOC patients

**Fig. 3:**
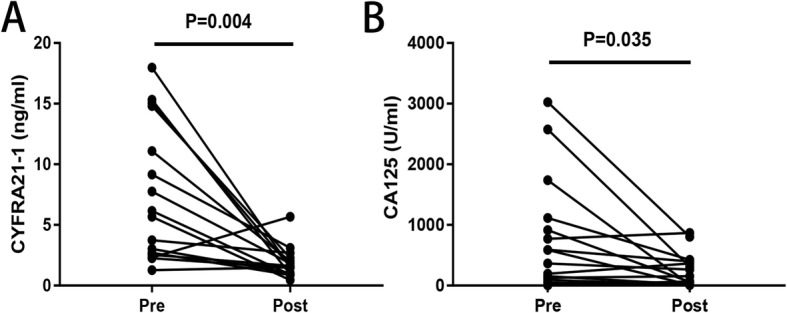
**a** Line chart of pre- and postoperative serum CYFRA21-1 expression. **b** Line chart of pre- and postoperative serum CA125 expression

Dynamic detections of serum CYFRA21-1 and CA125 were carried out in 17 patients with EOC before and after surgery. Figure 3 shows that serum CYFRA21-1 and CA125 levels after surgery were significantly lower than those before treatment (*P* < 0.05). There was a general trend that CYFRA21-1 along with CA125 was significantly higher before surgery and decreased progressively in the follow-up period after surgery. An elevation may indicate recurrence or metastasis.

### Correlations between serum CYFRA21-1 and OS of EOC patients

**Fig. 4:**
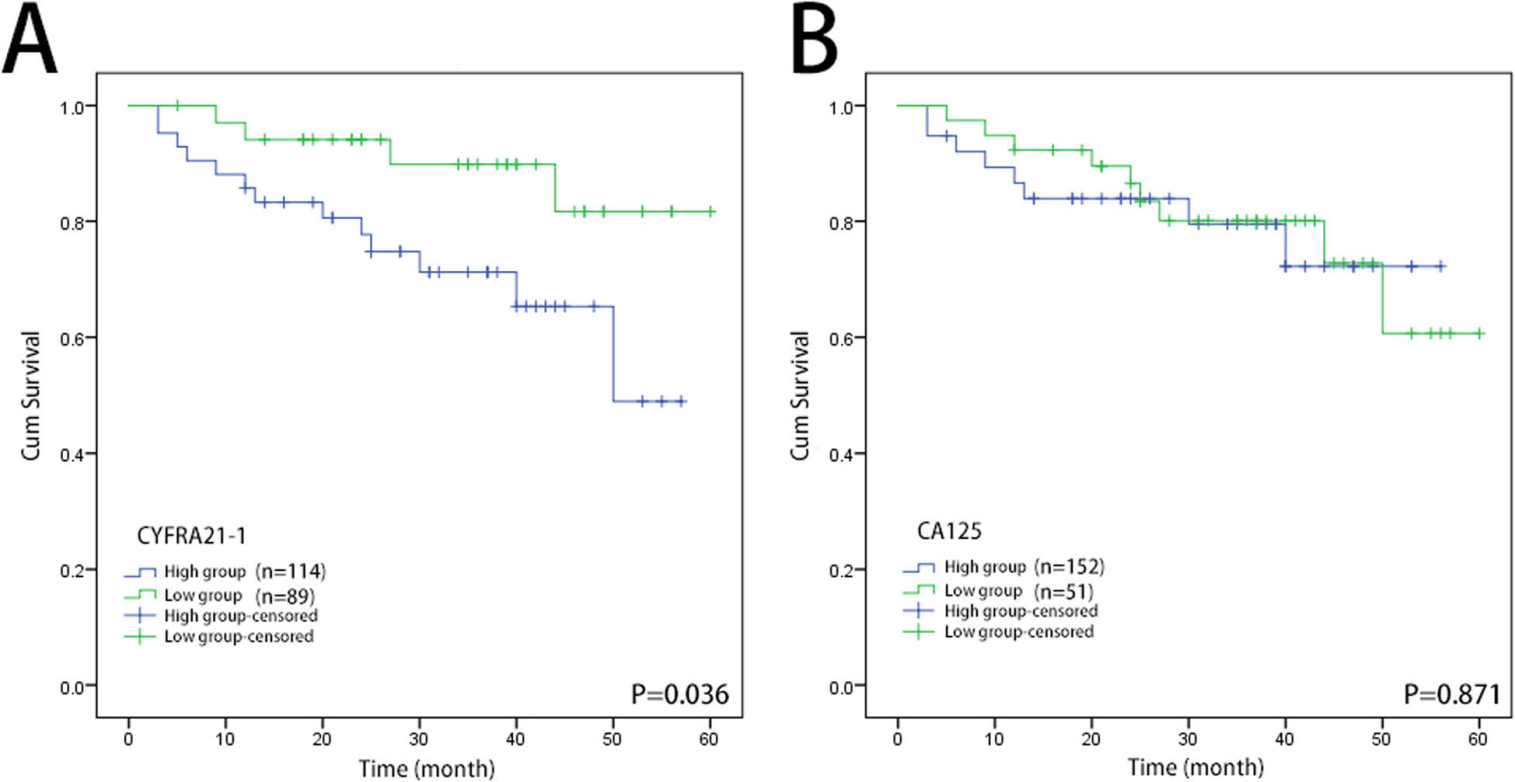
**a** Kaplan-Meier curve of CYFRA21-1 for the overall survival of 203 patients with epithelial ovarian cancer (EOC). **b** Kaplan-Meier curve of CA125 for the overall survival of 203 patients with epithelial ovarian cancer (EOC)

Kaplan-Meier survival analysis and log-rank tests were determined according to the postoperative survival time to evaluate the prognostic value of CYFRA21-1. From the Kaplan-Meier curve, patients with higher CYFRA21-1 expression displayed a shorter overall survival rate than those with lower CYFRA21-1 expression (Fig. 4a, log rank test, *P* < 0.05). The median survival time of the high CYFRA21-1 group was 42 months (95% confidence interval (CI): 36.384–49.026 months) and 54 months in the low CYFRA21-1 group (95% confidence interval (CI): 49.246–59.493 months) (χ^2^ = 4.417, *P* = 0.036). However, there was no significant difference between the high CA125 group and the low CA125 group (Fig. 4b). The median survival time of the high CA125 group was 45 months (95% confidence interval (CI): 39.758–52.093 months) and 49 months in the low CA125 group (95% confidence interval (CI): 43.327–55.266 months) (χ^2^ = 0.026, *P* = 0.871). These results indicated that CYFRA21-1 may act as a prognostic biomarker of EOC.

### Univariate and multivariate analyses for the prognosis of EOC patients

Univariate analysis and multivariate analyses were performed by a Cox proportional hazards regression model to further assess the prognostic value of CYFRA21-1. In the univariate analysis, tumor grade (*P* = 0.050), tumor stage (*P* = 0.004) and CYFRA21-1 (*P* = 0.047) were associated with OS **(**Table 4**)**. However, CA125 did not have a significant correlation with OS (*P* = 0.538). In multivariate analysis, the influence of CYFRA21-1 on OS (hazard ratio = 1.094, 95% CI = 0.307–3.897; *P* = 0.890) was lost, while tumor stage remained (hazard ratio = 2.086 95% CI = 1.115–3.901; *P* = 0.021). Hence, our data may indicate that CYFRA21-1 was not an independent prognostic marker for EOC.

**Table 4:** Univariate and multivariate analyses of prognostic factors on OS by the Cox regression model

Variables	Univariate analysis		multivariate analysis			
	HR	95% CI of HR	*p* value	HR	95% CI of HR	*p* value
Age	1.361	0.520–3.558	0.530	1.776		
Tumor size	0.821	0.266–2.529	0.731			
Subtype	0.194	0.032–1.185	0.076			
Grade	3.65	1.002–13.290	0.050*	2.952	0.816–10.681	0.099
Stage	2.22	1.289–3.821	0.004*	2.086	1.1115–3.901	0.021*
Lymph node	2.049	0.779–5.391	0.146			
Ascites	2.874	0.785–10.518	0.111			
CA125	1.42	0.463–4.370	0.538			
CYFRA21-1	3.128	1.017–9.624	0.047*	1.094	0.307–3.897	0.890

*P < 0.05

## Discussion

Ovarian cancer is the most lethal gynecologic malignancy among women living in industrialized countries. Ovarian cancer is one of the most frequent invasive female genital tract malignancies, with an estimated 22,240 cases diagnosed annually in the USA [14]. A total of 52,100 cases and 22,500 deaths occurred annually in China [15]. Genetic, hormonal, reproductive, environmental, and ethnic factors increase risk [16]. Ovarian cancer is not a single-disease entity but rather comprises a heterogeneous group of tumors with distinct clinicopathological characteristics [17–19]. The different histological types of ovarian cancer have a distinct biology and clinical behavior. However, previous studies have not considered biologic heterogeneity. In this article, we deliberately selected epithelial ovarian cancer (EOC) as our study object to prevent the interference of heterogeneous factors.

Currently, there are many new diagnostic markers for ovarian cancer, such as mesothelin [20], TRIM44 [21] and BCRA1 methylation [22]. However, they are not perfect and have a high cost and low reproducibility. CA125 is the classical marker but is not specific for EOC; it can be detected in other benign gynecologic disorders and other cancers, such as endometrial and pancreatic cancers [4, 9]. Nearly 6% of women without ovarian cancer CA125 levels gained more than 35 U/ml [6, 23]. Moreover, mucinous EOC and clear cell cancer do not express CA125 or express little [18, 24]. According to research by Buys SS et al., screening with CA125 and TVU simultaneously did not reduce ovarian cancer mortality compared with usual care [25]. Furthermore, it has been proven that screening for ovarian cancer can lead to unnecessary surgical harm for healthy women [26].

Cytokeratins constitute the main component of keratin filaments and are key parts of the cell cytoskeleton [27]. Cytokeratin 19 (CK19) represents the type I acidic cytokeratins that are expressed in many epithelial malignancies, such as lung cancer, breast cancer, cervical carcinoma, colorectal carcinoma and papillary thyroid carcinoma [28, 29]. The main functions of CK19 are maintaining the integrity of epithelial cell and participating in the immune response [19, 30]. CYFRA21-1 is the cytokeratin 19 fragment that is released into the serum from epithelial cells during the late S and G2 phases [13]. CYFRA21-1 has been known as a hopeful biomarker for many cancers, and its overexpression has been detected in lung cancer, colorectal cancer [31] and bladder cancer [32], especially in squamous cell carcinoma [33]. However, to our knowledge, few comparative studies have focused on the relationship between CYFRA21-1 and EOC.

In the present study, we found that CA125 is superior to CYFRA21-1 in the diagnosis of EOC. Preoperative levels of CYFRA21-1 and CA125 were significantly higher in EOC patients than in the benign group and normal control. However, there were no significant changes in the CYFRA21-1 level between the benign group and the normal control, while the CA125 level was significantly different. However, the preoperative level of CYFRA21-1 is a good complement for CA125 to improve the diagnostic sensitivity and negative predictive value. In the prognostic aspect, Kaplan-Meier survival analysis showed a significant difference in median survival time between the high CYFRA21-1 group and the low CYFRA21-1 group, while CA125 was not significantly different between the two groups. Univariable analysis showed that CYFRA21-1, grade, and stage were all significantly associated with OS (Table 3). However, CA125 was not the influencing factor of OS. CA125 and its optimal usage in EOC prognosis has been controversial [6, 7, 18]. Some scholars believe that serum CA125 has no clinical value for the follow-up monitoring of postoperative patients with EOC, which was consistent with our study (33). Thus, CYFRA 21-1 is likely to be superior to CA125 as a prognostic indicator in EOC.

This study highlights that the serum CYFRA21-1 level may be a useful noninvasive biomarker in monitoring EOC patients. We found that higher serum CYFRA21-1 levels in patients with EOC were significantly correlated with stage, grade, lymph node metastasis and ascites, thus confirming the engagement of CYFRA21-1 in tumor invasion. Taken together, our study demonstrated that CYFRA21-1 could be valuable in EOC diagnosis and prognosis. Patients with high CYFRA 21-1 levels should be followed up frequently to avoid undesirable consequences.

This study is a retrospective, single-center, small-sample study with a single source of research objects (all from the Affiliated Hospital of Nantong University). The benign group has limited types of lesions, while the healthy group is only the staff of physical examination in our hospital, which has certain limitations. However, it has advantages in that it is a relatively large patient group and consistent treatment. Further prospective multicenter studies will be needed to provide more definitive data to clarify the significance of our findings.

## Conclusion

In summary, preoperative serum CYFRA 21-1 levels have been shown to have a tight relationship with poor grade, advanced stage, ascites and lymph metastasis. We demonstrated the potential value of the serum CYFRA21-1 level as a complementary diagnostic tool for EOC in conjunction with CA125. Additionally, CYFRA 21-1 is likely to be superior to CA125 as a prognostic indicator in EOC.

## Data Availability

Data sharing is not applicable to this article as no datasets were generated or analysed during the current study.

## References

[1] Montagnana M, Benati M, Danese E (2017). Circulating biomarkers in epithelial ovarian cancer diagnosis: from present to future perspective. Ann Transl Med.

[2] Cliby WA, Powell MA, Al-Hammadi N, Chen L, Philip Miller J, Roland PY (2015). Ovarian cancer in the United States: contemporary patterns of care associated with improved survival. Gynecol Oncol.

[3] Edgell T, Martin-Roussety G, Barker G, Autelitano DJ, Allen D, Grant P (2010). Phase II biomarker trial of a multimarker diagnostic for ovarian cancer. J Cancer Res Clin Oncol.

[4] Gloss BS, Samimi G (2014). Epigenetic biomarkers in epithelial ovarian cancer. Cancer Lett.

[5] Jacob F, Meier M, Caduff R, Goldstein D, Pochechueva T, Hacker N (2011). No benefit from combining HE4 and CA125 as ovarian tumor markers in a clinical setting. Gynecol Oncol.

[6] Pradjatmo H (2016). Impact of preoperative serum levels of CA 125 on epithelial ovarian cancer survival. Asian Pac J Cancer Prev.

[7] Guo N, Peng Z (2017). Does serum CA125 have clinical value for follow-up monitoring of postoperative patients with epithelial ovarian cancer? Results of a 12-year study. J Ovarian Res.

[8] Felder M, Kapur A, Gonzalez-Bosquet J, Horibata S, Heintz J, Albrecht R (2014). MUC16 (CA125): tumor biomarker to cancer therapy, a work in progress. Mol Cancer.

[9] Granato T, Porpora MG, Longo F, Angeloni A, Manganaro L, Anastasi E (2015). HE4 in the differential diagnosis of ovarian masses. Clin Chim Acta.

[10] Van Calster B, Timmerman D, Bourne T, Testa AC, Van Holsbeke C, Domali E (2007). Discrimination between benign and malignant adnexal masses by specialist ultrasound examination versus serum CA-125. J Natl Cancer Inst.

[11] Barillo JL, da Silva Junior CT, Silva PS, de Souza JBS, Kanaan S, Xavier AR (2018). Increased cytokeratin 19 fragment levels are positively correlated with adenosine deaminase activity in malignant pleural effusions from adenocarcinomas. Dis Markers.

[12] Xie Y, Zhang Y, Du L, Jiang X, Yan S, Duan W (2018). Circulating long noncoding RNA act as potential novel biomarkers for diagnosis and prognosis of non-small cell lung cancer. Mol Oncol.

[13] Wu HH, Wang PH, Yeh JY, Chen YJ, Yen MS, Huang RL (2014). Serum cytokeratin-19 fragment (Cyfra 21-1) is a prognostic indicator for epithelial ovarian cancer. Taiwan J Obstet Gynecol.

[14] Smith RA, Andrews KS, Brooks D, Fedewa SA, Manassaram-Baptiste D, Saslow D (2018). Cancer screening in the United States, 2018: a review of current American Cancer Society guidelines and current issues in cancer screening. CA Cancer J Clin.

[15] Chen W, Zheng R, Baade PD, Zhang S, Zeng H, Bray F (2016). Cancer statistics in China, 2015. CA Cancer J Clin.

[16] Jelovac D, Armstrong DK (2011). Recent progress in the diagnosis and treatment of ovarian cancer. CA Cancer J Clin.

[17] Kurman RJ, Shih Ie M (2011). Molecular pathogenesis and extraovarian origin of epithelial ovarian cancer--shifting the paradigm. Hum Pathol.

[18] Chen X, Zhang J, Cheng W, Chang DY, Huang J, Wang X (2013). CA-125 level as a prognostic indicator in type I and type II epithelial ovarian cancer. Int J Gynecol Cancer.

[19] Rojas V, Hirshfield KM, Ganesan S, Rodriguez-Rodriguez L (2016). Molecular characterization of epithelial ovarian cancer: implications for diagnosis and treatment. Int J Mol Sci.

[20] Okła K, Surówka J, Frąszczak K, Czerwonka A, Kaławaj K, Wawruszak A (2018). Assessment of the clinicopathological relevance of mesothelin level in plasma, peritoneal fluid, and tumor tissue of epithelial ovarian cancer patients. Tumor Biol.

[21] Liu S, Yin H, Ji H, Zhu J, Ma R (2018). Overexpression of TRIM44 is an independent marker for predicting poor prognosis in epithelial ovarian cancer. Exp Ther Med.

[22] Chaudhry P, Srinivasan R, Patel FD (2009). Utility of gene promoter methylation in prediction of response to platinum-based chemotherapy in epithelial ovarian cancer (EOC). Cancer Investig.

[23] Wang V, Li C, Lin M, Welch W, Bell D, Wong Y-F (2005). Ovarian cancer is a heterogeneous disease. Cancer Genet Cytogenet.

[24] Lu D, Kuhn E, Bristow RE, Giuntoli RL, Kjaer SK, Shih Ie M (2011). Comparison of candidate serologic markers for type I and type II ovarian cancer. Gynecol Oncol.

[25] Buys SS, Partridge E, Black A, Johnson CC, Lamerato L, Isaacs C (2011). Effect of screening on ovarian cancer mortality: the prostate, lung, colorectal and ovarian (PLCO) cancer screening randomized controlled trial. JAMA.

[26] Grossman DC, Curry SJ, Owens DK, Barry MJ, Davidson KW, U.S.P.S.T. Force (2018). Screening for ovarian cancer: US preventive services task force recommendation statement. JAMA.

[27] Moll R, Franke WW, Schiller DL, Geiger B, Krepler R (1982). The catalog of human cytokeratins: patterns of expression in normal epithelia, tumors and cultured cells. Cell.

[28] Mehrpouya M, Pourhashem Z, Yardehnavi N, Oladnabi M (2019). Evaluation of cytokeratin 19 as a prognostic tumoral and metastatic marker with focus on improved detection methods. J Cell Physiol.

[29] Park SY, Lee JG, Kim J, Park Y, Lee SK, Bae MK (2013). Preoperative serum CYFRA 21-1 level as a prognostic factor in surgically treated adenocarcinoma of lung. Lung Cancer.

[30] Karantza V (2011). Keratins in health and cancer: more than mere epithelial cell markers. Oncogene.

[31] Thomas DS, Fourkala EO, Apostolidou S, Gunu R, Ryan A, Jacobs I (2015). Evaluation of serum CEA, CYFRA21-1 and CA125 for the early detection of colorectal cancer using longitudinal preclinical samples. Br J Cancer.

[32] Miyake M, Morizawa Y, Hori S, Tatsumi Y, Onishi S, Owari T (2017). Diagnostic and prognostic role of urinary collagens in primary human bladder cancer. Cancer Sci.

[33] Liu L, Liu B, Zhu LL, Li Y (2013). CYFRA21-1 as a serum tumor marker for follow-up patients with squamous cell lung carcinoma and oropharynx squamous cell carcinoma. Biomark Med.

